# Diagnostic Accuracy of NICE Classification System for Optical Recognition of Predictive Morphology of Colorectal Polyps

**DOI:** 10.1155/2018/7531368

**Published:** 2018-03-14

**Authors:** Josipa Patrun, Lucija Okreša, Hrvoje Iveković, Nadan Rustemović

**Affiliations:** ^1^School of Medicine, University of Zagreb, Zagreb, Croatia; ^2^Department of Gastroenterology and Hepatology, University Hospital Centre Zagreb, Zagreb, Croatia

## Abstract

The NICE classification is an international endoscopic classification of colorectal neoplasia through a narrowband spectrum that on the basis of lesion colors, vascular pattern, and structure of the surface of the mucous membrane classifies colorectal neoplasms in three categories: type 1 as hyperplastic lesions, type 2 as adenomas, and type 3 as invasive tumors. The aim of this study was to verify diagnostic accuracy of the NICE classification system compared to the reference standard: histopathological analysis. This retrospective study was conducted by ten physicians on a sequential sample of 418 patients and 735 polyps. The total diagnostic accuracy of the NICE classification system is found to be 76.7%. Optical recognition is significantly better with larger polyps, high-risk lesions (HGIEN), and neoplastic lesions. This research has shown that the NICE classification system is at the moment inferior to histopathological analysis. However, it is noticed that some physicians achieve significantly better results, with the accuracy of diagnosis ranging from 59.5% to 84.2%. These results show that with proper training of physicians and the use of endoscope enhancements to improve image quality, the NICE classification system could in the future potentially replace the histopathological diagnosis process.

## 1. Introduction

Colon cancer is a malignant tumor that arises from epithelial cells of a colon and is the most common malignant tumor of a digestive system [1]. It is ranked third among the most common malignant tumors in men (746,000.0; 10%) and second in women (614,000.0; 9.2%) in the world, while in Croatia, it is the second cause of mortality from malignant diseases in men and women (in 2013, 1182 males and 855 females died) [2]. In women, it is ranked second in the incidence (1261 newly discovered cases in 2012), and in men, it is ranked third (1700 newly discovered cases in 2012). According to [2], epidemiological data clearly indicate that colorectal cancer is one of the most important public health issues in Croatia, and its growing incidence and mortality rates warn the importance of multidisciplinary approaches, ensuring availability of new treatment modalities when needed, as well as insisting on secondary and primary prevention (e.g., population-based screening, changes in dietary habits and physical levels, and education of the public). The golden standard of contemporary gastrointestinal practice in the detection and removal of colorectal polyps today is colonoscopy. Colonoscopy is a diagnostic tool aimed for visualizing of the colon mucosa and for detecting colorectal polyps and colorectal cancer. It has been accepted as the most effective method for screening colon neoplasms in patients older than 50 years and younger patients with elevated risk for cancer [3].

Also, colonoscopy is a method by which we can intervene therapeutically. The fact is that the colonoscopy with polypectomy successfully prevents the occurrence of colorectal cancer, which reduces the mortality caused by this disease [4]. It provides adequate material for histopathological analysis which contains valuable data on the type of neoplasia, the degree of dysplasia and, in the case of a malignant neoplasm, about its possible invasion in the submucosa and lymph vessels. Among newly discovered polyps, very small (≤5 mm) and small (6–10 mm) polyps are dominant [5–7]. It is known that these polyps have initially low malignant potential (tubular adenomas and sedimentary serum polyps), as well as that hyperplastic polyps have no tendency to malignant transformations [5, 8]. Removal of all newly discovered polyps (including those with little or no probability of malignant transformation) creates additional costs to the health system, and patients are exposed to the risk of polypectomy (bleeding, perforation of the intestine) that can be avoided [7, 9]. The ability to distinguish nonneoplastic from neoplastic polyps in vivo would allow selective removal of polyps, as well as selective transmission to histopathological analysis. Therefore, in the last two decades, many techniques of so-called optical and digital chromoendoscopy have been developed, which do not require direct coloring of tissues, and instead are based on optical filters and digital image processing that emulate a classical chromoendoscopic image. Narrow-band imaging (NBI) is one of the methods of optical chromoendoscopy that uses high-resolution colonoscopy to give a detailed description of the surface tissue of the neoplasm, as well as its vascular pattern and thus characterizes the polyps while performing endoscopy (in vivo), so-called virtual biopsy, which would be an equivalent to the histological nomenclature [10, 11]. Diagnosis based on angiogenesis or vascular morphological change can be ideal for early detection and diagnosis of neoplastic lesions, as angiogenesis plays a critical role in the transition of premalignant lesions in a hyperproliferative state to a malignant phenotype [12]. In this way, it is possible, at the moment of discovery of the neoplasm, to decide on the course of further treatment (endoscopic resection or surgical procedure), as well as on the patient follow-up period, unlike the standard gastrointestinal endoscopic practice where decision is made after the histopathological analysis. The international endoscopic classification of colorectal neoplasms with narrowband spectrum (NICE) has been developed and it classifies colorectal neoplasia in three categories based on three characteristics (color of lesions, vascular pattern, and lining surface): type 1 are hyperplastic lesions, type 2 are adenomas, and type 3 are invasive carcinomas [13, 14]. Type 1 lesions should only be monitored, type 2 lesions should be polypectomied, and type 3 lesions should be removed endoscopically, if possible (endoscopic mucosectomy or endoscopic submucosal dissection), or with surgical intervention.

The aim of this study was to evaluate the reliability of virtual biopsy of colorectal polyps in routine colonoscopy practice. The specific goal was to determine the accuracy, sensitivity, and narrowband spectrum specificity in the evaluation of polyp histology using the NICE classification.

## 2. Materials and Methods

The research was retrospectively performed at the Department of Gastroenterology and Hepatology of the Clinic for Internal Diseases of University Hospital Centre Zagreb. In the study, we included all patients with polypectomy of diminutive (1–5 mm), small (6–9 mm), large (10–20 mm), and extra-large (>20 mm) colon polyps in the period from July 2015 to July 2016, which were previously characterized by narrowband spectrum using the NICE classifications. A consecutive sample of all patients examined during the follow-up period was selected. The sample consisted of 418 patients and 735 polyps, where the examinations were performed by 10 UHC Zagreb physicians. A type of the endoscope used in this study is Olympus EXERA III (CF-HQ190L/l) video colonoscope. The physicians, who set the diagnosis based on optical recognition of the polyps' histology, were blinded to the results of the histopathological analysis. Location, size, and shape according to the Paris classification [15] were determined and recorded for each polyp. The findings of the histopathological analysis of the removed polyps, classified according to the European Quality Assurance Guidelines for Screening and Diagnosis of CRC [16], were recorded and compared to a specific type of lesion according to the NICE classification. The study did not include patients for whom the results of histopathological analysis were not available, or the material was artificially changed and could not be analyzed. The research was conducted in accordance with the ethical principles of the 1975 Helsinki Declaration of the World Health Organization and their 1983 amendments [17]. The identity of the patients was hidden. The research was not reported on the public register. The protocol of research was approved by the Ethics Committee of UHC Zagreb. The target population included in this study consisted of patients of both sexes, who were subjected to colonoscopy for suspected colorectal polyps. Analysis of the required sample size and statistical strength of the study was not performed before the data collection has started, as it was decided to include all the patients who were screened in the follow-up period.

## 3. Results and Discussion

### 3.1. Data Analysis

This chapter provides quantitative and statistical analysis of the collected data and finally a discussion of the obtained results. The level of statistical significance in all statistical tests is set to 0.05 (*α* = 0.05) and all confidence intervals (CIs) to 95%. In the analysis of a correlation of individual characteristics of patients and polyps with diagnostic accuracy, two-tailed tests of statistical significance were used. The statistical analysis was made by using the statistical software package: NCSS 10 Statistical Software (2015; NCSS, LLC; Kaysville, Utah, USA).

### 3.2. Quantitative Data Analysis

**Figure 1:**
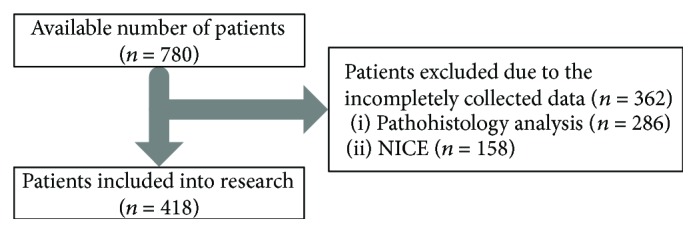
Number of patients involved in the study analysis.

**Figure 2:**
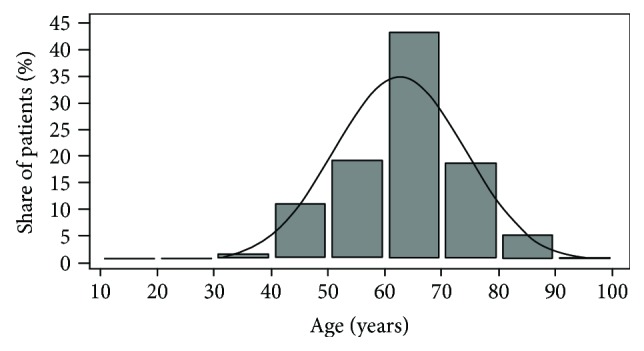
Age distribution of patients involved in the study (*n* = 418); the dotted line represents theoretical normal distribution with the empirically obtained arithmetic mean age.

During the observed period, 780 patients underwent colonoscopy due to suspected colorectal polyps. Data on histological analysis were correctly collected for 494 patients, and the NICE classification was properly made for 622 patients. Finally, 418 patients who had the valid data on the both key variables were included in the study (Figure 1). The study included 250 (59.8%) men and 168 (40.2%) women with age from 17 to 90 years.  Figure 2 shows the distribution of the number of patients involved in the study by age, with a median of 63 years. Detailed numerical data about the patients included in the study are given in Table 1, where the patients are divided into the categories related to patients' age and sex, number of patients per each physician, and number of analyzed polyps per patient.

**Figure 3:**
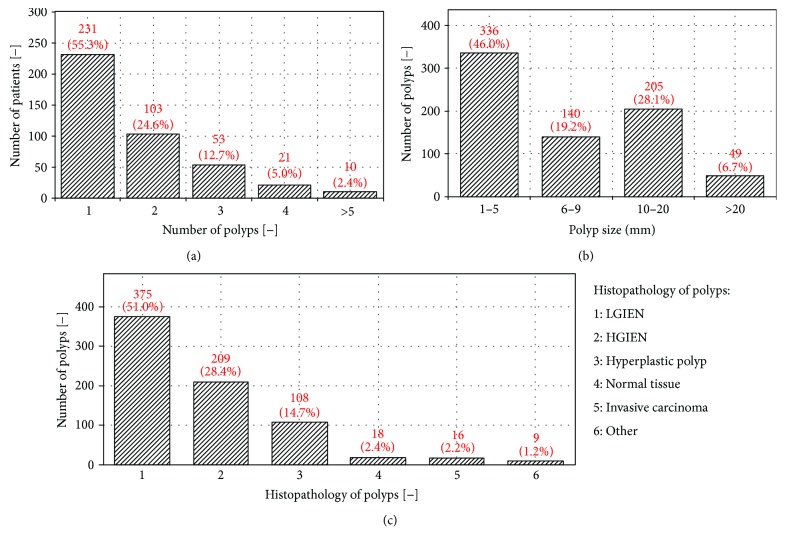
Distribution of number of patients over number of polyps per patient (a), number of polyps over size (b), and of number of polyps over histopathology analysis (1–6) (c).

The total number of polyps for the examined 418 patients for which the histopathological and the NICE classification results were available was 735. The number of patients categorized by the number of polyps is shown in Figure 3(a), where 55.3% patients had just one polyp, and this number decreases with the number of polyps per patient (more than five polyps had 2.4% of the patients examined). The polyps are subdivided by size into four categories: diminutive (1–5 mm), small (6–10 mm), large (10–20 mm), and very large (>20 mm), where 46.0% polyps were diminutive and 6.7% polyps were very large (Figure 3(b)). The median polyp size is 6 mm. Furthermore, the distribution of polyps by histopathological categories is shown in Figure 3(c), where the largest number of polyps are in the LGIEN (low-grade intraepithelial neoplasia) and HGIEN (high-grade intraepithelial neoplasia) categories (79.4%) corresponding to NICE 2 type, the lower number of polyps is in the category of hyperplastic polyps (14.7%) corresponding to NICE 1 type, and the smallest number of polyps is in the category of invasive carcinoma (2.2%) corresponding to the NICE 3 type. Detailed numerical data related to the characteristics of polyps are given in Table 2, where the polyps are divided into the following categories: patients' age and sex, number of examined polyps by physician, polyps' location in a colon, size, the Paris endoscopic classification, the NICE classification, types and risks of lesions, and histopathological diagnosis.

### 3.3. Statistical Data Analysis

**Figure 4:**
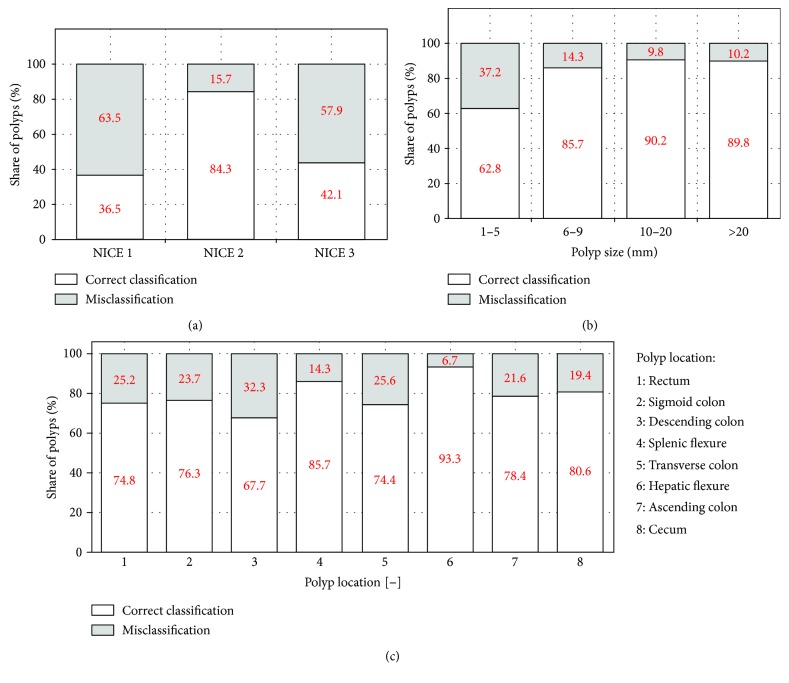
Share of correct classification and misclassification over different NICE types (a), different polyp sizes (b), and different polyp locations (1–8) (c).

Comprehensive numerical data related to the diagnostic accuracy of the NICE classification are given in Table 3, and the corresponding data analysis is given as follows. Total diagnostic accuracy of the NICE classification system for optical recognition of colorectal polyps' histology was 76.7%, with a 95% confidence interval ranging from 73.6% to 79.6%.  Figure 4(a) shows the distribution of classification accuracy over the NICE types, where it can be seen that the share of accurately classified polyps is highest in the case of polyps classified as NICE 2 (84.3%), whereas that portion is significantly lower for polyps classified as NICE 1 (36.5%) and NICE 3 (42.1%). However, since the largest number of polyps is classified as NICE 2 type, it contributes most to the overall accuracy of the result. Furthermore, Figure 4(b) shows the distribution of correct classification per polyp size, where it can be seen that the lowest accuracy of the classification is 62.8% in the case of the diminutive polyps, while the accuracy of classification increases by a polyp size and is about 90% for large and very large polyps.  Figure 4(c) shows the distribution of accurate classification for individual colon parts which rates from 67.7% in descending colon to 93.3% in hepatic flexure.

The diagnostic accuracy of the NICE classification is further analyzed by using the statistical chi-squared (*χ*^2^) test. Thus, the accuracy of the classification for two groups of considered polyps is statistically significantly different if the corresponding *p* value (or corrected *p*′; the last two columns in Table 3) is smaller than the level of significance *α* = 0.05. Correspondingly, the total accuracy of the NICE classification was statistically significantly different for different polyp sizes (small, large, and very large when compared to diminutive), for pedunculated (Ip) and flat elevations of mucosa (IIa) compared to sessile polyps (Is), for neoplastic compared to nonneoplastic lesions, and high-risk compared to low-risk lesions (see *p* and *p*′ values in Table 3). In addition to *p* and *p*′ values, Table 3 also includes OR (odds ratio) values that indicate the relative accuracy of the considered polyp group when compared to the reference group with OR = 1 (in the case of OR ≥ 1 the classification accuracy of the considered polyps' group is higher for OR value times than the classification accuracy of the reference group, while the case of OR < 1 only means it is lower than in the case of reference one). Therefore, the accuracy of the classification was significantly better for pedunculated polyps (Ip) by 2.65 times (OR = 2.65) and significantly lower in flat elevations of mucosa (IIa) (OR = 0.38), when compared to sessile polyps (Is). For neoplastic lesions, classification accuracy is 8.7 times higher than in nonneoplastic lesions (OR = 8.70), and 3.81 times higher (OR = 3.81) in high-risk lesions (HGEIN, invasive carcinoma) than in low-risk lesions (LGIEN, hyperplastic, normal, and other). In comparison to the diminutive polyps (1–5 mm), the small polyps (6–9 mm) had 3.56 times higher probabilities for accurate diagnosis (OR = 3.56, 95% CI: 2.11–5.99, *p* < 0.001), while large (10–20 mm) had 5.48 times higher probability for accurate diagnosis (OR = 5.48; 95% CI: 3.29–9.14; *p* < 0.001).

**Figure 5:**
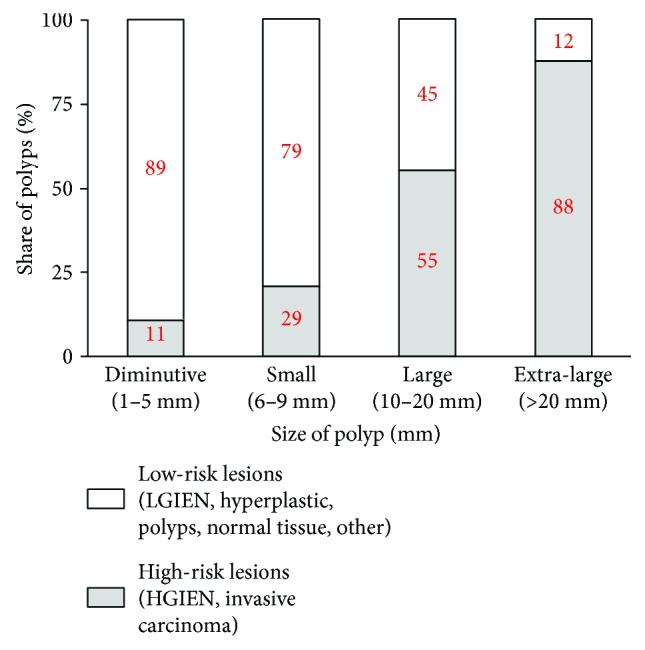
Distribution of the risk of the polyps over different groups of polyp sizes.

Figure 5 shows the distribution of the risk of the polyps by different polyp sizes from which it can be concluded that the proportion of high-risk polyps is consistent and clinically relevant with the polyp size. This is further confirmed by the statistical chi-squared test which shows that this difference is statistically significant (*χ*^2^ = 203.6; number of degrees of freedom = 3; *p* < 0.001). The value of Cramer's V, which represents the correlation of two considered categorical variables (in this case polyps and risk), equals to 0.53, which can be considered a fair correlation (the maximum correlation would be in the case of Cramer's V = 1, while the minimum correlation would be in the case of Cramer's V = 0).

**Figure 6:**
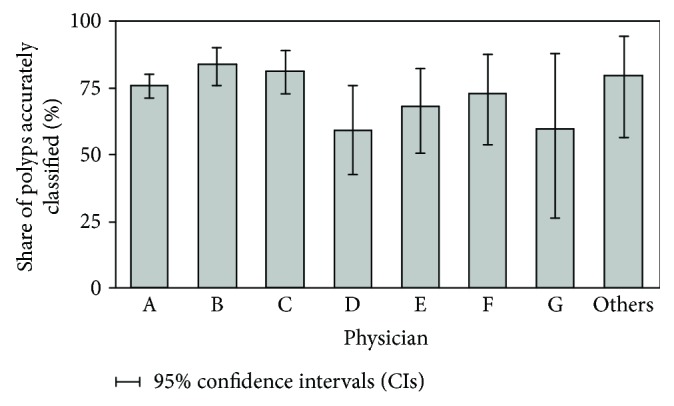
Diagnostic accuracy over different physicians along with the 95% confidence intervals.

Furthermore, there is a difference in the accuracy of diagnostics between physicians, where the accuracy ranged from 59.5% (physician D) to 84.2% (physician B) (Figure 6). However, the reliability of these results depends significantly on the number of polyps evaluated and classified by the individual physician so that the smaller number of evaluated polyps, in this case, means less reliable results, which is manifested in a wider 95% confidence intervals (Table 3, Figure 6). Three physicians (physicians A, B, and C) treated a significantly higher number of polyps than other physicians, and therefore, their results are much more reliable (narrower confidence intervals). In addition, they also have a higher accuracy of the diagnosis (76.3%, 84.2%, and 81.6%), suggesting that the accuracy of the physician's diagnosis may be improved by the number of performed polyp classifications.

In Table 4, a detailed analysis of the accuracy of the NICE classification by types is given. The table rows refer to the NICE type assigned by the physician (predicted), while the table columns indicate the NICE type derived from histopathological analysis (reference: hyperplastic polyps→NICE 1; LGIEN, HGIEN→NICE 2; invasive cancer→NICE 3). The first row of the table indicates the distribution of the number of polyps classified by physicians as NICE 1 over reference categories obtained from histopathological analysis. Thus, most of the polyps classified as NICE 1 were actually NICE 2 (52.9%), while the smaller number were correctly classified (36.5%). Significant number of incorrect NICE 1 classifications were mistaken for normal tissue (9.6%) or other nonpolyp lesions (1%). Also, in the case of polyps classified as NICE 2, the highest number is correctly classified (84.3%), while the smaller part actually falls into the NICE 1 category (11.4%). Among the polyps classified as NICE 3, the major part is actually NICE 2 (57.9%), while 42.1% of polyps were correctly classified.

### 3.4. Discussion

If the margin of noninferiority to the histopathological analysis, which is meant to be the benchmark with the 100% accuracy, is set to 20%, the entire 95% confidence interval of the total accuracy of optical recognition was below the margin of noninferiority. However, the accuracy was noninferior to the histopathological analysis (the whole 95% confidence interval above the margin of noninferiority) in the case of large polyps (10–20 mm), peduncular (Ip) polyps according to the Paris endoscopic classification, type 2 polyps according to the NICE classification, neoplastic lesions, and LGIEN and HGIEN polyps. In other polyp categories, lower classification accuracy is noted, diminutive polyps with 62.8% (95% CI 57.4–68.0) and nonneoplastic lesions with 36.5% accuracy (95% CI 27.3–46.5). These results point to polyp categories whose features and specificities could be further explored and, in line with new findings, educate physicians to ultimately improve the overall diagnostic accuracy of optical recognition.

Also, among the physicians performing the NICE classification, three physicians (Table 3, physicians A, B, and C) had the highest accuracy of 76.3%, 84.2%, and 81.6%. It should be noted that these physicians had also the highest number of classifications, so their results are the most reliable which is reflected in the narrowest 95% confidence intervals. This finding implies that the classification accuracy could be dependent on number of classifications performed, which would then point to the importance of frequent training.

Table 4 shows which polyp categories physicians mistaken the most, where it can be seen that NICE 1 and NICE 2 types, and NICE 2 and NICE 3 types are frequently mistaken between each other. NICE 1 type polyps do not require medical intervention (polypectomy), NICE 2 type polyps require polypectomy and monitoring, while NICE 3 category requires urgent treatment. According to Table 4 (NICE 1 column), it can be seen that 70 out of 108 polyps (64.8%), NICE 1 polyps are misclassified into NICE 2, that is, from a group that does not require polypectomy in the group that requires it. Only 35.2% polyps were correctly classified as NICE 1. This type of error can increase the cost of treatment due to the unnecessary analysis of higher number of polyps. Furthermore, polyps that are actually NICE 2 type (2nd column in Table 4) were classified as NICE 1 to a lower extent (55 out of 582, 9.4%), i.e., from a group requiring polypectomy in a group that does not require it, what represents a certain risk for a patient if untreated. Very small portion of NICE 2 polyps are classified as NICE 3 polyps (11 out of 582, 1.9%), while they are mostly correctly classified (516 out of 582, 88.7%). These results point to the share of misclassifications that lead to increased treatment costs and to the share of health risks that should be taken into account when planning future training and education of physicians.

The average accuracy of the NICE classifications presented in this paper of 76.7% is lower when compared to the similar research given in [13], where 96% of classification accuracy is achieved, while it is in line with large multicentric study conducted in the UK [18], concluding that NBI optical diagnostics are currently not recommended in routine clinical practice (the achieved accuracy of NBI optical diagnostics was 83.4%). The reasons for different levels of accuracy can be multiple, for example, related to the level of expertise of physicians who performed classifications, colonoscopies, equipment used, and so forth. The related discussion is given as follows. Physicians should pass through more frequent trainings in the recognition of pathologic mucosal and vascular specimens of colorectal polyps' mucosa. This applies both to experts and inexperienced physicians who are conducting endoscopies. In this study, a number of examined patients and polyps per physician in the considered time period of one year, given in Tables 1 and 2, reflect the experience of each physician in the NICE classification, since they had passed the training in the NICE classification right before the start of that period. It can be seen that these numbers vary significantly, from 5 to 223 patients and from 10 to 388 polyps per physician. Furthermore, the quality of colonoscopies should be strictly controlled in order to ensure the same conditions for all examination samples [19]. More precisely, this means that the share of cecum intubation, the share of adenoma detection, and the preprocedural colon cleansing should be in accordance with the standards established by the Endoscopic Section of Croatian Gastroenterology Association. Another important part which could impact NICE classification accuracy are endoscopic instruments. It is important that all instruments have the ability to increase an image with “near (dual) focus.” Also, it is important to use the “cap” at the top of an endoscope with the aim to stabilize and optimize an endoscopic image to a target position and to persistently purge the polyps (e.g., by using acetylcysteine), until all mucus and faeces are removed and thus making the image clear. This is supported by a large number of different solutions and cleaning regimes that are available today for cleansing the bowels [8]. A magnifying endoscope was used only on a small and insignificant number of patients included in this study. However, it is expected that the usage of magnifying endoscope would help physicians in polyps' classifications, especially in the case of diminutive lesions, and thus further contribute to the improvement of the obtained classification accuracy. Furthermore, there is a problem when a single polyp has patterns with characteristics of different NICE types. In these cases, it is recommended to physicians to classify the analyzed polyp to a higher NICE class (e.g., if the polyp contains patterns of both NICE 1 and NICE 2 types, then the whole polyp should be classified as NICE 2). However, in this research, the part of physicians have classified the polyps with different patterns into more than one type, for example, if a polyp has both characteristics of NICE 1 and NICE 2, or NICE 2 and NICE 3, it is classified as NICE 1/2 or NICE 2/3, respectively, which have made a statistical analysis more difficult and which have certainly decreased the total accuracy of classification to some extent. For the sake of future research studies, it is recommended that physicians decide and classify each analyzed polyp into only one class out of three possible (without intermediate classes), and without classifying polyps to higher NICE classes if polyps have mixed patterns, when performing colonoscopy and determining the NICE classification.

This research has indicated the potential of replacing the histopathological diagnosis process with the NICE classification system of colorectal polyps' histology, although the current classification accuracy is not at the target level. The research has shown that additional expert work is needed in establishing clear rules for recognition and graduation of pathological patterns and more frequent endoscopic trainings for physicians (e.g., every 6 months if possible). However, we need to be aware that the NICE classification goes through further validation process and that this research is also a new and important part of the whole process.

## 4. Conclusion

Colorectal cancer (CRC) is the most common malignant tumor of the digestive system and the second cause of mortality from malignant diseases in Croatia. Since the histopathological analysis of each removed polyp is time consuming and expensive, new methods, such as the NICE (narrow-band imaging international colorectal endoscopic) classification of colorectal neoplasms, are very valuable. Some research suggest that NBI-assisted optical diagnostics has acceptable accuracy to determine the diagnosis without histopathological analysis. However, to introduce it in clinical practice, additional studies should be carried out, and the effectiveness of this method should be verified on a large number of patients.

The main results of this study show that overall accuracy of polyp diagnosis was 76.7%. These results range from about 60 to 85% depending on the physician who has given the diagnosis, indicating that the accuracy of the diagnosis depends, to a substantial extent, on the physician performing the procedure. Furthermore, it is shown that in the case of small polyps (6–9 mm), the appearance of accurate diagnosis is approximately three times greater than in the case of a diminutive (1–5 mm) polyps (OR = 3.56), while in the case of large polyps (10–20 mm), accurate diagnosis is more than five times larger (OR = 5.48). Also, a statistical test showed that the risk of lesion was statistically significantly related to polyp size. Currently, the diagnostic accuracy is not sufficient to apply this diagnostic method in routine clinical practice.

The main contributions of this paper are in (a) quantifying the accuracy of diagnosis by the NICE classification on a relatively large number of patients and polyps; (b) observing that the accuracy of the diagnosis varies greatly depending on the physician who has given the diagnosis, suggesting that the education and training of the physician could improve the accuracy of the diagnosis; and (c) observing that misclassification predominantly happens between NICE 1 and NICE 2 polyps' types, where classification of actual NICE 1 (according to histopathology) to NICE 2 type happens more frequently; misclassifications between NICE 1 and NICE 3 polyps' types and vice versa are not perceived; and actual NICE 3 polyps are very frequently mixed up with NICE 2 polyps (in 50% situations).

Additionally, the accuracy of the diagnosis could be improved by using endoscope supplements to improve image quality, such as plastic caps placed on top of the endoscope and therefore provide stable visualization of the sample, and certainly insisting on perfect visual field cleaning. It should also be noted that the value of this research is that it is the first in the Republic of Croatia that deals with the application of the NICE classification system in clinical practice and represent the base for further related research. It is expected that this research will encourage endoscopists in other centers to collaborate to further validate this valuable method and ultimately implement it into routine clinical practice.

## Figures and Tables

**Table 1:** Characteristics of patients included in the study (*n* = 418).

	*n*	(%)
Sex		
Male	250	(59.8)
Female	168	(40.2)
Age (years), median (IQR)	63	(57–69)
Age (years)		
≤44	31	(7.4)
45–54	53	(12.7)
55–64	156	(37.3)
65–74	130	(31.1)
≥75	48	(11.5)
Age (years) by sex, median (IQR)		
Male	63	(58–69)
Female	63	(55–70)
Number of examined patients per physician		
Physician A	223	(53.3)
Physician B	64	(15.3)
Physician C	56	(13.4)
Physician D	24	(5.7)
Physician E	16	(3.8)
Physician F	14	(3.3)
Physician G	5	(1.2)
Other physicians	16	(3.8)
Patients age per physician (years), median (IQR)		
Physician A	63	(55–69)
Physician B	65	(61–70)
Physician C	62	(58–70)
Physician D	62	(55–67)
Physician E	66	(62–75)
Physician F	61	(54–72)
Physician G	72	(66–80)
Other physicians	54	(44–68)
Number of analyzed polyps per patient		
1	231	(55.3)
2	103	(24.6)
3	53	(12.7)
4	21	(5.0)
5	7	(1.7)
6	2	(0.5)
10	1	(0.2)

The data is presented as the number (percentage), unless otherwise indicated; IQR: interquartile range—measure of statistical dispersion, being equal to the difference between 75th and 25th percentiles or between upper and lower quartiles.

**Table 2:** Characteristics of polyps included in the study (*n* = 418 patients; *n* = 735 polyps).

	*n*	(%)
Sex of the patient		
Male	490	(66.7)
Female	245	(33.3)
Patients age (years), median (IQR)	63	(58–70)
Patients age (years)		
≤44	40	(5.4)
45–54	81	(11.0)
55–64	291	(39.6)
65–74	220	(29.9)
≥75	103	(14.0)
Age (years) by sex, median (IQR)		
Male	63	(59–70)
Female	63	(56–70)
Number of examined polyps per physician		
Physician A	388	(52.8)
Physician B	114	(15.5)
Physician C	98	(13.3)
Physician D	37	(5.0)
Physician E	38	(5.2)
Physician F	30	(4.1)
Physician G	10	(1.4)
Other physicians	20	(2.7)
Polyp location		
Rectum	115	(15.6)
Sigmoid colon	320	(43.5)
Descending colon	65	(8.8)
Splenic flexure	7	(1.0)
Transverse colon	39	(5.3)
Hepatic flexure	30	(4.1)
Ascending colon	51	(6.9)
Cecum	108	(14.7)
Polyp size (mm), median (IQR)	6	(4–12)
Polyp size (mm)		
Diminutive (1–5 mm)	336	(46.0)
Small (6–9 mm)	140	(19.2)
Large (10–20 mm)	205	(28.1)
Extra-large (>20 mm)	49	(6.7)
The Paris endoscopic classification		
Sessile polyps (Is)	497	(69.5)
Pedunculated polyps (Ip)	137	(19.2)
Subpedunculated polyps (Is + Ip)	44	(6.2)
Flat elevation of mucosa (IIa)	27	(3.8)
Flat elevation with central depression (IIa + IIc)	5	(0.7)
IIa + IIb	3	(0.4)
Is + IIa	2	(0.3)
The NICE classification		
1	104	(14.1)
2	612	(83.3)
3	19	(2.6)
Lesions (NICE classification)		
Nonneoplastic	104	(14.1)
Neoplastic	631	(85.9)
Histopathological diagnosis		
LGIEN	375	(51)
HGIEN	209	(28.4)
Hyperplastic polyp	108	(14.7)
Normal tissue	18	(2.4)
Invasive carcinoma	16	(2.2)
Other	9	(1.2)
The risk of lesions		
Low (LGIEN, hyperplastic, normal tissue, and other)	510	(69.4)
High (HGEIN, invasive carcinoma)	225	(30.6)

The data is presented as the number (percentage), unless otherwise indicated. The data were not correctly collected for the Paris endoscopic classification for 20 (2.7%) patients.

**Table 3:** Diagnostic accuracy of the NICE classification system for optical recognition of colorectal polyps histology (*n* denotes number of polyps).

		Share of accurate diagnosis					
	*n*	*n*	(%)	95% CI	OR	*p*	*p*′
All polyps	735	564	(76.7)	(73.4–79.7)			
Sex of the patient							
Male	490	368	(75.1)	(71.0–78.9)	1		
Female	245	196	(80.0)	(74.4–84.8)	1.33	0.139	>0.999
Age of patients (years)							
≤44	40	34	(85.0)	(70.2–94.3)	1		
45–54	81	57	(70.4)	(59.2–80.0)	0.42	0.085	>0.999
55–64	291	223	(76.6)	(71.3–81.3)	0.58	0.238	>0.999
65–74	220	169	(76.8)	(70.7–82.2)	0.59	0.254	>0.999
≥75	103	81	(78.6)	(69.4–86.1)	0.65	0.392	>0.999
Number of examined polyps per physician							
Physician A	388	296	(76.3)	(71.7–80.4)	1		
Physician B	114	96	(84.2)	(76.2–90.4)	1.66	0.074	>0.999
Physician C	98	80	(81.6)	(72.5–88.7)	1.38	0.260	>0.999
Physician D	37	22	(59.5)	(41.8–75.0)	0.46	0.027	0.567
Physician E	38	26	(68.4)	(51.3–82.5)	0.67	0.284	>0.999
Physician F	30	22	(73.3)	(54.1–87.7)	0.86	0.715	>0.999
Physician G	10	6	(60.0)	(26.2–87.8)	0.47	0.245	>0.999
Other physicians	20	16	(80.0)	(56.3–94.3)	1.24	0.703	>0.999
Polyp location							>0.999
Rectum	115	86	(74.8)	(65.9–82.4)	1		>0.999
Sigmoid colon	320	244	(76.3)	(71.3–80.9)	1.08	0.752	>0.999
Descending colon	65	44	(67.7)	(55.0–78.8)	0.71	0.309	>0.999
Splenic flexure	7	6	(85.7)	(42.1–99.6)	2.02	0.522	>0.999
Transverse colon	39	29	(74.4)	(57.9–87.0)	0.98	0.958	0.958
Hepatic flexure	30	28	(93.3)	(77.9–99.2)	4.72	0.042	0.840
Ascending colon	51	40	(78.4)	(64.6–88.7)	1.23	0.612	>0.999
Cecum	108	87	(80.6)	(71.9–87.6)	1.40	0.303	>0.999
Polyp size (mm)							
Diminutive (1–5 mm)	336	211	(62.8)	(57.4–68.0)	1		
Small (6–9 mm)	140	120	(85.7)	(78.9–91.0)	3.56	<0.001	<0.001
Large (10–20 mm)	205	185	(90.2)	(85.3–93.9)	5.48	<0.001	<0.001
Extra-large (>20 mm)	49	44	(89.8)	(77.8–96.6)	5.21	0.001	0.025
The Paris endoscopic classification							
Sessile polyps (Is)	497	368	(74.0)	(69.9–77.8)	1		
Pedunculated polyps (Ip)	137	121	(88.3)	(81.7–93.2)	2.65	0.001	0.024
Subpedunculated polyps (Is + Ip)	44	37	(84.1)	(69.9–93.4)	1.85	0.146	>0.999
Flat elevation of mucosa (IIa)	27	14	(51.9)	(32.0–71.4)	0.38	0.015	0.345
Flat elevation with central depression (IIa + IIc)	5	5	(100.0)	(47.8–100)	-		
IIa + Iib	3	2	(66.7)	(9.4–99.2)	-		
Is + Iia	2	2	(100.0)	(15.8–100)	-		
The NICE classification							
1	104	38	(36.5)	(27.3–46.5)	1		
2	612	516	(84.3)	(81.2–87.1)	0.34	<0.001	<0.001
3	19	8	(42.1)	(28.8–75.5)	1.93	0.191	>0.999
Lesions (NICE classification)							
Nonneoplastic	104	38	(36.5)	(27.3–46.5)	1		
Neoplastic	631	526	(83.4)	(80.3–86.2)	8.70	<0.001	<0.001
Histopathological diagnosis							
LGIEN	375	325	(86.7)	(82.8–90.0)	1		
HGIEN	209	195	(93.3)	(89.9–96.3)	2.14	0.016	0.352
Hyperplastic polyp	108	36	(33.3)	(24.5–43.0)	0.08	<0.001	<0.001
Normal tissue	18	0	(0.0)	(0.0–18.5)	0.00		
Invasive carcinoma	16	8	(50.0)	(24.7–75.4)	0.15	<0.001	<0.001
Other	9	0	(0.0)	(0.0–33.6)	0.00		
The risk of lesions							
Low (LGIEN, hyperplastic, normal tissue, and other)	510	361	(70.8)	(66.6–74.7)	1		
High (HGEIN, invasive carcinoma)	225	203	(90.2)	(85.5–93.8)	3.81	<0.001	<0.001

The data is presented as the number (percent), unless otherwise indicated. 1 = reference category; OR = odds ratio; 95% CI = 95% confidence interval; *p* = statistical significance of appearance for accurate diagnosis; *p*′ = statistical significance corrected by sequential Holm-Bonferroni correction; - = statistics have not been calculated because of the low polyp count.

**Table 4:** Confusion matrix distribution of the NICE classifications performed by physicians (predicted) over the NICE classifications obtained from histopathology (reference).

		Reference (obtained from histopathology)					
		NICE 1	NICE 2	NICE 3	Normal	Other	∑
Predicted	NICE 1	38 (36.5%)	55 (52.9%)	0 (0.0%)	10 (9.6%)	1 (1%)	104 (100%)
	NICE 2	70 (11.4%)	516 (84.3%)	8 (1.3%)	10 (1.6%)	8 (1.3%)	612 (100%)
	NICE 3	0 (0.0%)	11 (57.9%)	8 (42.1%)	0 (0.0%)	0 (0.0%)	19 (100%)
	∑	108	582	16	20	9	735

Percentages shown within brackets represent shares of polyps classified by physicians (predicted) among the reference classes. Alternatively, the shares of polyps in percentages can be expressed for the reference classes over the predicted ones (see Discussion).

## References

[1] Damjanov I., Seiwerth S., Jukić S., Nola M. (2014). *Patologija*.

[2] Šekerija M., Marković T. (2015). Epidemiology of colorectal cancer in Croatia and worldwide. *Medical Sciences*.

[3] Rex D. K., Petrini J. L., Baron T. H. (2006). Quality indicators for colonoscopy. *The American Journal of Gastroenterology*.

[4] Winawer S. J., Zauber A. G., Ho M. N. (1993). Prevention of colorectal cancer by colonoscopic polypectomy. *New England Journal of Medicine*.

[5] Rex D. K., Overhiser A. J., Chen S. C., Cummings O. W., Ulbright T. M. (2009). Estimation of impact of American College of Radiology recommendations on CT colonography reporting for resection of high-risk adenoma findings. *The American Journal of Gastroenterology*.

[6] Lieberman D., Moravec M., Holub J., Michaels I., Eisen G. (2008). Polyp size and advanced histology in patients undergoing colonoscopy screening: implications for CT colonography. *Gastroenterology*.

[7] Hassan C., Pickhardt P. J., Rex D. K. (2010). A resect and discard strategy would improve cost-effectiveness of colorectal cancer screening. *Clinical Gastroenterology and Hepatology*.

[8] Hassan C., Pickhardt P. J., Kim D. H. (2009). Systematic review: distribution of advanced neoplasia according to polyp size at screening colonoscopy. *Alimentary Pharmacology & Therapeutics*.

[9] Rabeneck L., Paszat L. F., Hilsden R. J. (2008). Bleeding and perforation after outpatient colonoscopy and their risk factors in usual clinical practice. *Gastroenterology*.

[10] Rastogi A., Keighley J., Singh V. (2009). High accuracy of narrow band imaging without magnification for the real-time characterization of polyp histology and its comparison with high-definition white light colonoscopy: a prospective study. *The American Journal of Gastroenterology*.

[11] Gono K., Obi T., Yamaguchi M. (2004). Appearance of enhanced tissue features in narrow-band endoscopic imaging. *Journal of Biomedical Optics*.

[12] Iwatate M., Ikumoto T., Hattori S., Sano W., Sano Y., Fujimori T. (2012). NBI and NBI combined with magnifying colonoscopy. *Diagnostic and Therapeutic Endoscopy*.

[13] Hewett D. G., Kaltenbach T., Sano Y. (2012). Validation of a simple classification system for endoscopic diagnosis of small colorectal polyps using narrow-band imaging. *Gastroenterology*.

[14] Hayashi N., Tanaka S., Hewett D. G. (2013). Endoscopic prediction of deep submucosal invasive carcinoma: validation of the narrow-band imaging international colorectal endoscopic (NICE) classification. *Gastrointestinal Endoscopy*.

[15] Participants in the Paris Workshop (2003). The Paris endoscopic classification of superficial neoplastic lesions: esophagus, stomach, and colon. *Gastrointestinal Endoscopy*.

[16] Aabakken L., Altenhofen L., Ancell-Park R., Antoljak N., Armatoli P., Arrossi S. (2014). *ur. Prijevod europskih smjernica za osiguranje kvalitete probira i dijagnostike raka debelog crijeva*.

[17] World Medical Association (2013). World Medical Association Declaration of Helsinki: ethical principles for medical research involving human subjects. *Journal of the American Medical Association*.

[18] Rees C. J., Rajasekhar P. T., Wilson A. (2016). Narrow band imaging optical diagnosis of small colorectal polyps in routine clinical practice: the Detect Inspect Characterise Resect and Discard 2 (DISCARD 2) study. *BMJ*.

[19] Pulanić R., Rustemović N., Bilić B. (2015). *Algoritmi u gastrointestinalnoj endoskopiji i endoskopskom ultrazvuku: kvaliteta u gastrointestinalnoj endoskopiji*.

